# Synchronous Brain Dynamics Establish Brief States of Communality in Distant Neuronal Populations

**DOI:** 10.1523/ENEURO.0005-21.2021

**Published:** 2021-05-06

**Authors:** Martin Seeber, Christoph M. Michel

**Affiliations:** 1 Functional Brain Mapping Lab, Department of Fundamental Neuroscience, Campus Biotech, University of Geneva, Geneva 1202, Switzerland; 2 Center for Biomedical Imaging (CIBM), Lausanne and Geneva, Lausanne 1015, Switzerland

**Keywords:** EEG source imaging, electroencephalography, large-scale functional networks, resting state, simultaneous synchronization, spatial leakage problem

## Abstract

Intrinsic brain dynamics co-fluctuate between distant regions in an organized manner during rest, establishing large-scale functional networks. We investigate these brain dynamics on a millisecond time scale by focusing on electroencephalographic (EEG) source analyses. While synchrony is thought of as a neuronal mechanism grouping distant neuronal populations into assemblies, the relevance of simultaneous zero-lag synchronization between brain areas in humans remains largely unexplored. This negligence is because of the confound of volume conduction, leading inherently to temporal dependencies of source estimates derived from scalp EEG [and magnetoencephalography (MEG)], referred to as spatial leakage. Here, we focus on the analyses of simultaneous, i.e., quasi zero-lag related, synchronization that cannot be explained by spatial leakage phenomenon. In eighteen subjects during rest with eyes closed, we provide evidence that first, simultaneous synchronization is present between distant brain areas and second, that this long-range synchronization is occurring in brief epochs, i.e., 54–80 ms. Simultaneous synchronization might signify the functional convergence of remote neuronal populations. Given the simultaneity of distant regions, these synchronization patterns might relate to the representation and maintenance, rather than processing of information. This long-range synchronization is briefly stable, not persistently, indicating flexible spatial reconfiguration pertaining to the establishment of particular, re-occurring states. Taken together, we suggest that the balance between temporal stability and spatial flexibility of long-range, simultaneous synchronization patterns is characteristic of the dynamic coordination of large-scale functional brain networks. As such, quasi zero-phase related EEG source fluctuations are physiologically meaningful if spatial leakage is considered appropriately.

## Significance Statement

Synchrony is suggested as a mechanism for coordinating distant neuronal populations. However, simultaneous (i.e., zero-lag) synchronization between remote brain regions in humans is difficult to demonstrate, because volume conduction in electroencephalographic (EEG)/magnetoencephalographic (MEG) recordings causes spurious zero-lag relations. Here, we investigate actual zero-lag relations and systematically compare them to the residual bias resulting from spatial smoothness of EEG source estimates. We indeed report simultaneous synchronization between distant brain regions. These synchronization patterns manifest variably in time. We suggest that simultaneous synchronization is relevant when studying the dynamic, large-scale functional architecture in humans.

## Introduction

Brain activity spontaneously fluctuates during rest, when no specific task is instructed. Intriguingly, these fluctuations are correlated between distant brain regions, forming large-scale functional networks that are assumed to reflect spontaneous information integration during internal mentation (Raichle et al., 2001; Greicius et al., 2003; Smith et al., 2009; Brookes et al., 2011; Engel et al., 2013), i.e., the basis of thinking. While functional magnetic resonance imaging (fMRI) was crucial for the discovery and investigation of resting-state networks, the low time resolution of BOLD variations does not allow us to study the neurophysiological mechanisms leading to these spontaneous co-fluctuations of spatially distinct brain areas. Intracranial local field potential recordings or scalp electroencephalography/magnetoencephalography (EEG/MEG) are adequate for this purpose, as they record neuronal activity at their inherent time-scale, i.e., in the millisecond range (Roelfsema et al., 1997; Miller et al., 2009; Baker et al., 2014; Fox et al., 2018; Vidaurre et al., 2018). Such studies revealed an essential key neuronal mechanism underlying information integration between different brain regions: synchrony (Singer, 1999; Varela et al., 2001). Many studies have demonstrated that neuronal synchronization between brain areas is an important mechanism for the coordination of neuronal processing in anatomically distributed neuronal circuits (Engel et al., 1991; Contreras and Steriade, 1996; Roelfsema et al., 1997; Destexhe et al., 1999; Womelsdorf et al., 2007). A fundamental question is whether synchronous co-fluctuations between areas are simultaneous or time-lagged (Engel et al., 1991; Contreras and Steriade, 1996; Roelfsema et al., 1997; Destexhe et al., 1999; Fries, 2005; Womelsdorf et al., 2007; Siegel et al., 2008; Bosman et al., 2012; Van Kerkoerle et al., 2014). Because of delays because of axonal conduction and synaptic transmission, time-lagged fluctuations are necessarily appearing when the activation of one region is causally related to the activation of the other region, i.e., when one area transfers information to the other. Simultaneity, on the other hand, indicates a gathering of different brain areas converging into a functional unit to collectively maintain certain information without causal interactions between them. Such communality can be established spontaneously by dynamic recurrent connections or can be driven by a pacemaker (e.g., the thalamus; Vicente et al., 2008; Gollo et al., 2014). Undoubtedly, both mechanisms (time-lagged and simultaneous fluctuations) take place in the brain to processes, integrate and maintain information, as numerous intracranial recordings in animals and humans have shown (Contreras and Steriade, 1996; Roelfsema et al., 1997; Womelsdorf et al., 2007; Siegel et al., 2008; Hipp et al., 2011). Unfortunately, simultaneous activity, which imposes zero-lag-related signals are primarily ignored in EEG/MEG network analyses to avoid spurious phase relations resulting from volume conduction (Nolte et al., 2004; Stam et al., 2007; Hipp et al., 2012; Marzetti et al., 2013; Colclough et al., 2015). EEG/MEG source reconstruction (Michel et al., 2004; Michel and Murray, 2012; He et al., 2018) is, to some extent, able to overturn volume conduction effects. However, the limited spatial resolution of EEG/MEG source reconstruction techniques leads to spurious temporal relations (Palva et al., 2018; He et al., 2019). To correct for these spatial leakage effects, orthogonalization of source signals is a standard method. However, this method also discards genuine simultaneous dynamics and therefore is insensitive to detect such.

In this work, we aim to investigate simultaneous synchronization, i.e., quasi zero-lag relations between distant brain areas using high-density EEG source imaging (Michel et al., 2004; Michel and Murray, 2012; He et al., 2018). To consider and correct for spatial leakage effects, we systematically compare actual with surrogate data having identical spatial properties in their source reconstruction.

In summary, we demonstrate that physiologically meaningful quasi zero-lag synchrony between distant brain areas exists that cannot be explained by spatial leakage phenomena. We suggest that brief epochs of simultaneous synchronization signify functional convergence of distant neuronal population dynamics into distinct re-occurring states.

## Materials and Methods

### EEG recordings

High-density EEG was recorded using an electrode net (Geodesic Sensor Net, Electrical Geodesics Inc.) consisting of 256 electrodes that are interconnected by thin rubber bands. Each electrode includes a small sponge soaked with saline water to establish direct electrical contact with the participants’ scalp. EEG was sampled at 1 kHz, referenced to the vertex.

Participants (*N* = 18, 30 ± 5 years, seven male) sat comfortably in an upright position in a darkened, electrically shielded room and were instructed to keep their eyes closed and relax for 4–6 min (5.42 ± 0.95) avoiding drowsiness. The local ethical committee, following the declaration of Helsinki, approved the study. Participants provided written, informed consent for their participation.

### EEG preprocessing

EEG recordings were bandpass filtered between 1 and 40 Hz offline, and electrodes covering cheeks and nape were excluded. Time epochs contaminated with apparent artifacts were marked and excluded from further analyses. Noisy or bad electrodes were excluded from independent component analysis (ICA; Jung et al., 2000), which was used to remove stereotypical artifact components containing saccades, eye blinks, and cardiac artifacts. Afterward, the initially excluded channels were spline interpolated in space, resulting in 204 channels. The recordings were re-referenced to the common average and down-sampled to 125 Hz for further analysis.

### EEG source imaging and functional network reconstruction

We applied EEG source reconstruction using forward models based on realistic head geometry and conductivity data with consideration of skull thickness, i.e., locally spherical model with anatomical constraints (LSMAC; Brunet et al., 2011; Michel and Brunet, 2019). The gray matter was defined based on the MNI anatomic template model. The inverse solution space consisted of 5004 points equally distributed in this gray matter volume. The linear distributed inverse solution LAURA (Grave de Peralta Menendez et al., 2004) was used to calculate the current density distribution for each solution point at each moment in time. Dipole orientations were set to the first left singular vector of the *xyz* (3D) components in the resolution matrix of each source pointing outside of the brain to avoid sign ambiguities.

**Figure 1.**
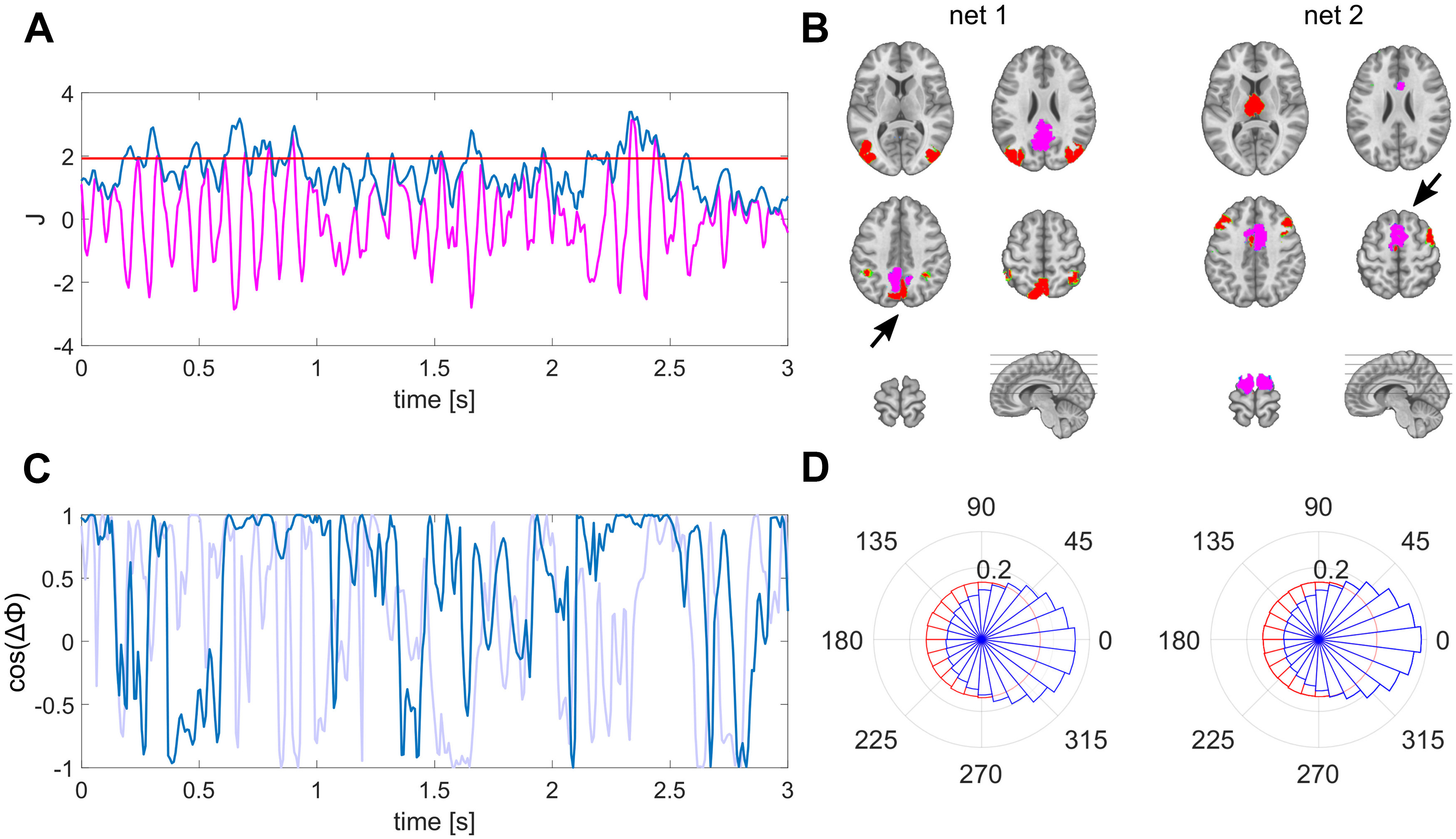
Derivation and characterization of EEG source reconstructed networks. ***A***, The envelope (blue) of source estimated activity (magenta) is thresholded to define periods of well-pronounced activity within a specific ROI (here PCC). ***B***, Nodes of the network (red) co-varying with the PCC (net 1) during periods defined as indicated in ***A*** and with the SMA (net 2) as seed ROI (magenta) marked with black arrows. ***C***, Exemplary time course of instantaneous phase locking between lateral posterior regions of net 1, matching the time period shown in ***A*** in magenta; surrogate phase locking is shown in light blue. ***D***, Polar histograms of the group, displaying the distribution of interhemispheric phase differences between lateral posterior (net 1) and anterior (net 2) regions as illustrated in ***B*** in blue; surrogate phase differences in red. The radius for each phase bin displays the probability density function estimate of the respective phase differences.

Functional networks were defined as spatial patterns co-varying with fluctuations in selected regions of interest (ROIs) defined in an atlas composed of 100 parcels (Schaefer et al., 2018). We chose the posterior cingulate cortex (PCC) and the supplementary motor area (SMA) as two exemplary seed regions based on previous literature focusing on functionally distinct key regions (Seeley et al., 2007; Raichle, 2010; Engel et al., 2013). The signal representing the activities in each ROI was defined as the first principal component of all dipoles within the given ROI (Rubega et al., 2019). Then, we calculated their signal envelope as the magnitude of the analytic signal using the Hilbert transform. To capture well-pronounced spatial patterns that include these key regions, we thresholded the signal envelope at the mean plus standard deviation following previous work (Tagliazucchi et al., 2012). The network patterns were then determined by sites that covary with this seed signal. To illustrate the resulting spatial patterns, they were spatially thresholded using watershed transform, and the local maxima positively co-varying with the respective ROI are shown (Fig. 1).

### Surrogate data and spatial leakage estimation

To systematically asses the bias introduced by spatial leakage we used surrogate data, which we derived from the actual data. To do so, we temporally shifted the source reconstructed signals of the actual data randomly in time for every solution point individually for each subject. These time shifts were randomly drawn without repeating numbers in the range of 1 and *N* samples, while *N* being the total number of samples. That way, the initial source dynamics of the surrogate data are the same as the actual source estimates, but the temporal relations between solution points are demolished. To introduce spatial leakage, we then applied the same forward model as used for analyzing actual data to generate surrogate EEG. Afterwards, we applied the identical processing pipeline to this surrogate data, i.e., filtering scalp data and source estimation using the same inversion kernel as in the analyses of the actual EEG data. Because we used identical forward model and inverse method for analyzing actual and surrogated data, the spatial properties of the source estimates are the same. That way, there are no actual correlations between the sources given the introduced random time shifts. Therefore, the resulting interareal correlation values in the surrogate source estimates are resulting from spatial leakage between selected areas. This procedure provides bias estimates caused by spatial leakage for every connectivity metric, i.e., correlation, phase-locking value (PLV) and coherence for each individual subject. These bias estimates can be subtracted from the metrics of actual data as suggested previously (Ghuman et al., 2011; Palva and Palva, 2012) and used for statistical comparison.

### Synchrony between network nodes

We investigated the correlation, lag, phase locking and coherence between network nodes. Between each pair, we determined the correlation for different lags of the signals using cross-correlation. To perform frequency-specific analyses, we applied wavelet transform (Morlet et al., 1982) for time-frequency (TF) decomposition (1–40 Hz, 1-Hz steps). Parameters for the mother wavelet were set to the full width at half maximum of 3 s for the Gaussian kernel at a center frequency of 1 Hz. At all frequencies, the number of cycles were kept constant meaning the Gaussian kernel of the wavelets was scaled accordingly, i.e., scale expansion factor of 1. PLV and coherence was computed for every frequency bin and are reported as magnitudes herein and for the latter as real and imaginary part of the coherency (Lachaux et al., 1999, 2002) to compare with previous literature (Nolte et al., 2004). Simultaneous synchrony is indicated as peak correlation at zero-lag in the cross-correlogram and the real part of coherency. The time-varying phase in each ROI was computed using Hilbert transform to determine phase differences between regions for every time point. The distribution of these phase differences was illustrated as polar histograms. The cosine of these phase differences ΔΦ was used as instantaneous measure of simultaneous synchronization, which is 1 for zero phase difference (Deco and Kringelbach, 2016; Cabral et al., 2017). The duration of phase synchrony, which is centered around zero phase lag was determined by epochs of cos(ΔΦ) exceeding 0.5. Very short epochs smaller than 24 ms, i.e., three time samples, were not considered as stable and therefore ignored for computing the average duration. All metrics were statistically compared with results derived from surrogate data. Paired comparisons were conducted using the Wilcoxon signed-rank test, which were Bonferroni corrected for multiple comparisons.

### Software accessibility

Code and data supporting the findings of this study are available on request to the corresponding author. EEG data analyses were performed using the freely available toolbox Cartool in combination with custom MATLAB scripts.

## Results

### Large-scale brain dynamics form briefly stable functional networks

We found bilateral, symmetric posterior regions in the extrastriate cortex and inferior parietal lobe (IPL) to co-vary with the PCC’s source signal. In contrast, we found anterior areas of the bilateral prefrontal cortex and the thalamus to co-vary with the SMA (Fig. 1*A*,*B*). To rule out a potential source imaging bias that might cause these patterns, we performed the same analyses on the surrogate data. Importantly, we found no distant spatial local maxima forming a network pattern in the surrogate data. Merely the respectively selected regions were present, meaning we did not observe co-varying regions using surrogate data (Fig. 6).

The phase relations between nodes of these functional network patterns in the real data vary considerably in time. We observe epochs in which the phase differences remain small, meaning these two nodes fluctuate synchronously at these time points (Fig. 1*C*,*D*). The durations of these epochs are in the range between 54.1 and 79.1 ms on average depending on the constellation. The durations of all pairs belonging to the same functional network are significantly longer than respective periods computed from surrogate data. The detailed duration of each pair and their respective *p* values are listed in Table 1.

**Table 1:** Correlation, PLV in the α range (8–12 Hz), probability density function estimate (pdf) at zero phase difference, and duration of each pair with respective *p* values (Wilcoxon sign-rank test, Bonferroni corrected)

	*r*	*p*_r_	PLV	*p*_PLV_	pdf_zero_	*p*_zero_	Dur (ms)	*p*_Dur_
Left IPL-PCC	0.20	0.0038	0.22	0.0011	0.24	0.0038	69.9	0.0007
Left IPL-right IPL	0.28	0.0007	0.28	0.0007	0.26	0.0007	79.1	0.0007
Right IPL-PCC	0.10	0.0123	0.15	0.0007	0.21	0.0322	66.2	0.0024
Left PFC-SMA	0.24	0.0012	0.34	0.0007	0.30	0.0017	57.1	0.0038
Left PFC-right PFC	0.31	0.0007	0.37	0.0007	0.29	0.0008	54.1	0.0020
Right PFC-SMA	0.26	0.0012	0.34	0.0007	0.32	0.0012	61.9	0.0009
SMA-thalamus	0.32	0.0020	0.34	0.0012	0.39	0.0014	75.8	0.0011

### Simultaneous synchronization is present between distant neuronal populations

**Figure 2.**
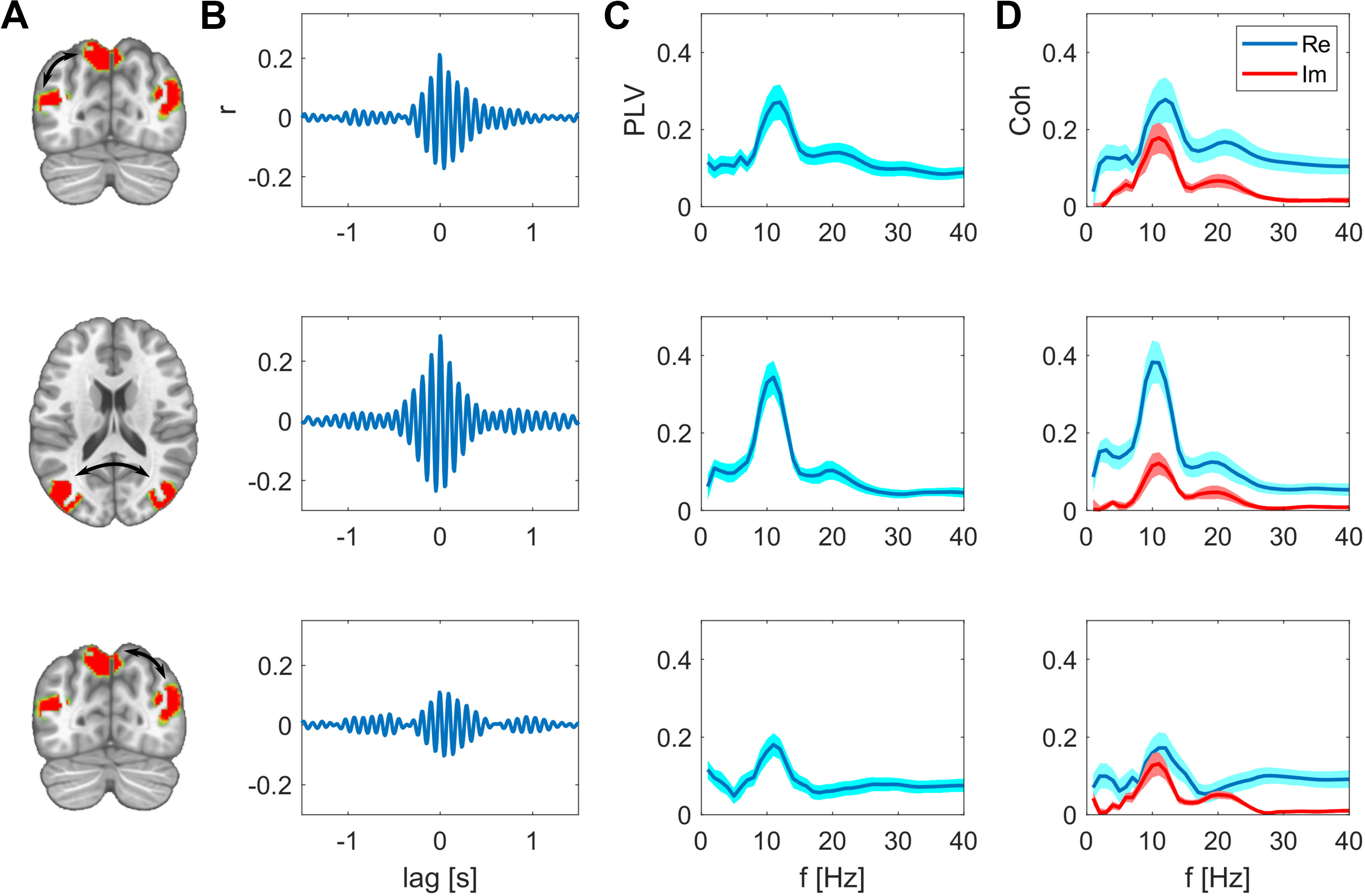
Synchrony between the nodes of the PPC network after subtracting spatial leakage bias. ***A***, Nodes of the network, edges are indicated as arrows. ***B***, Cross-correlations between these two nodes are respectively maximal at zero lag. ***C***, PLV as function of frequency, group mean ± SEM. ***D***, Real and imaginary part of the coherency, group mean ± SEM.

We identified functional network patterns that are composed of distinct nodes that are symmetric in both hemispheres (Fig. 1). This finding already indicates that these distant regions co-vary on a highly resolved time scale. To directly test whether the correlation between these nodes is significantly larger than the spatial leakage bias, we focused on the analyses of pairwise nodes for each network pattern. To provide more detail about these interactions, we investigated different time lags and frequency components. For the PCC-based network, we focused on posterior bilateral IPL regions. The cross-correlation between pairs of these network nodes peaks at zero-lag with values ranging between 0.1 and 0.28, which is significantly higher than the spatial leakage bias observed in the surrogate data. The detailed values are listed in Table 1. Interestingly, the interhemispheric zero-lag correlation was highest in this posterior network. The frequency-specific PLV reached its maximum for this pair at 11 Hz with a value of 0.34. In this case, the real part of the coherency is considerably higher than its imaginary part (Fig. 2).

**Figure 3.**
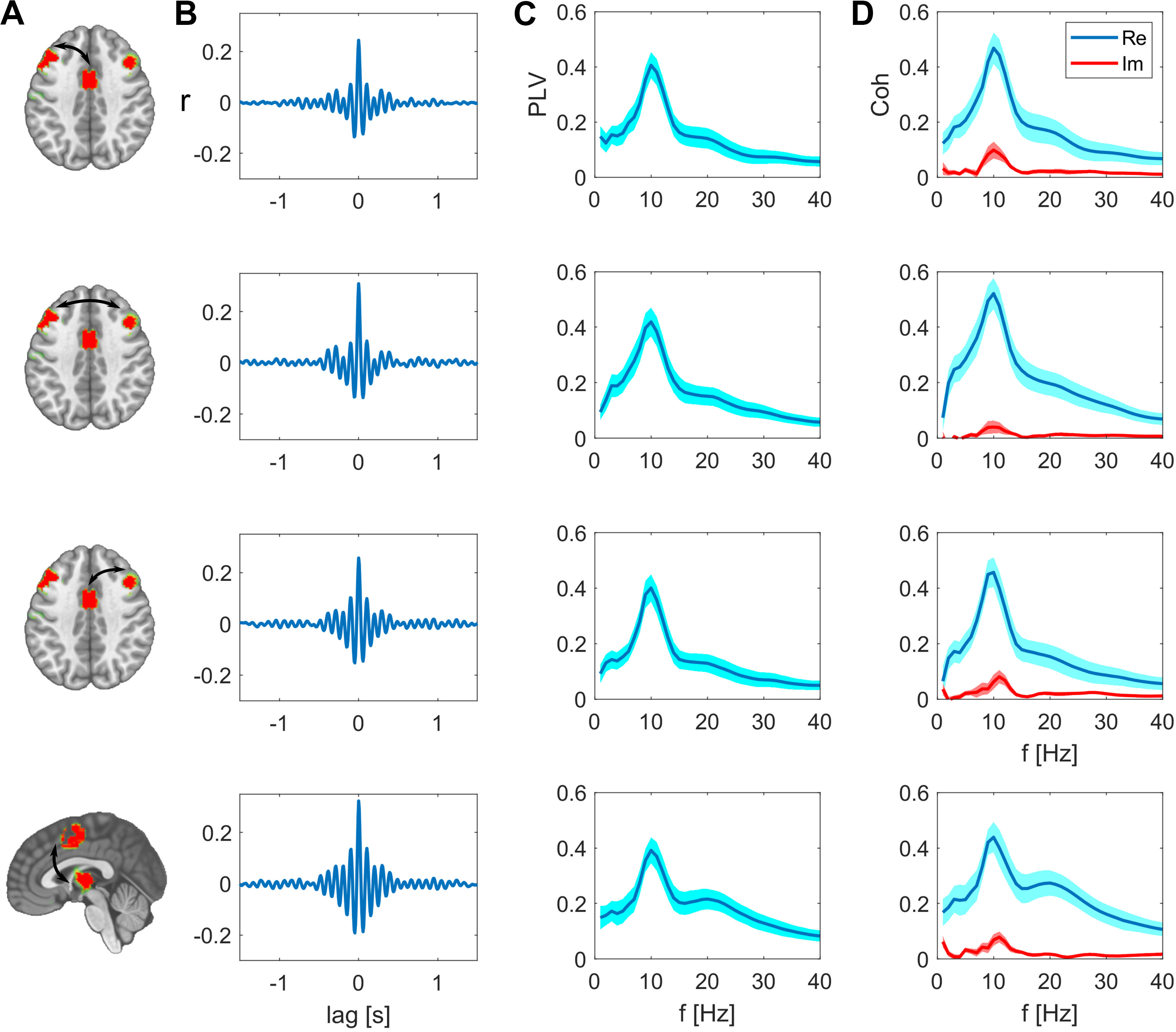
Synchrony between the nodes of the SMA network after subtracting spatial leakage bias. ***A***, Nodes of the network, edges are indicated as arrows. ***B***, Cross-correlations between these two nodes are respectively maximal at zero lag. ***C***, PLV as function of frequency, group mean ± SEM. ***D***, Real and imaginary part of the coherency, group mean ± SEM.

For the SMA-based network, we further examined the relation of the SMA to regions in the bilateral PFC and to the thalamus. The cross-correlation between these regions peaks at zero-lag with a value of ranging between 0.24 and 0.32, which is significantly higher than the spatial leakage bias observed in the surrogate data. The frequency-specific PLV reached its maximum at 10 Hz with a value of 0.42 for the interhemispheric PFC connection. Again, the real part of the coherency is higher than its imaginary part (Fig. 3). These results show that actual zero-phase relations, indicating simultaneous synchronization, are present between relatively distant regions.

**Figure 4.**
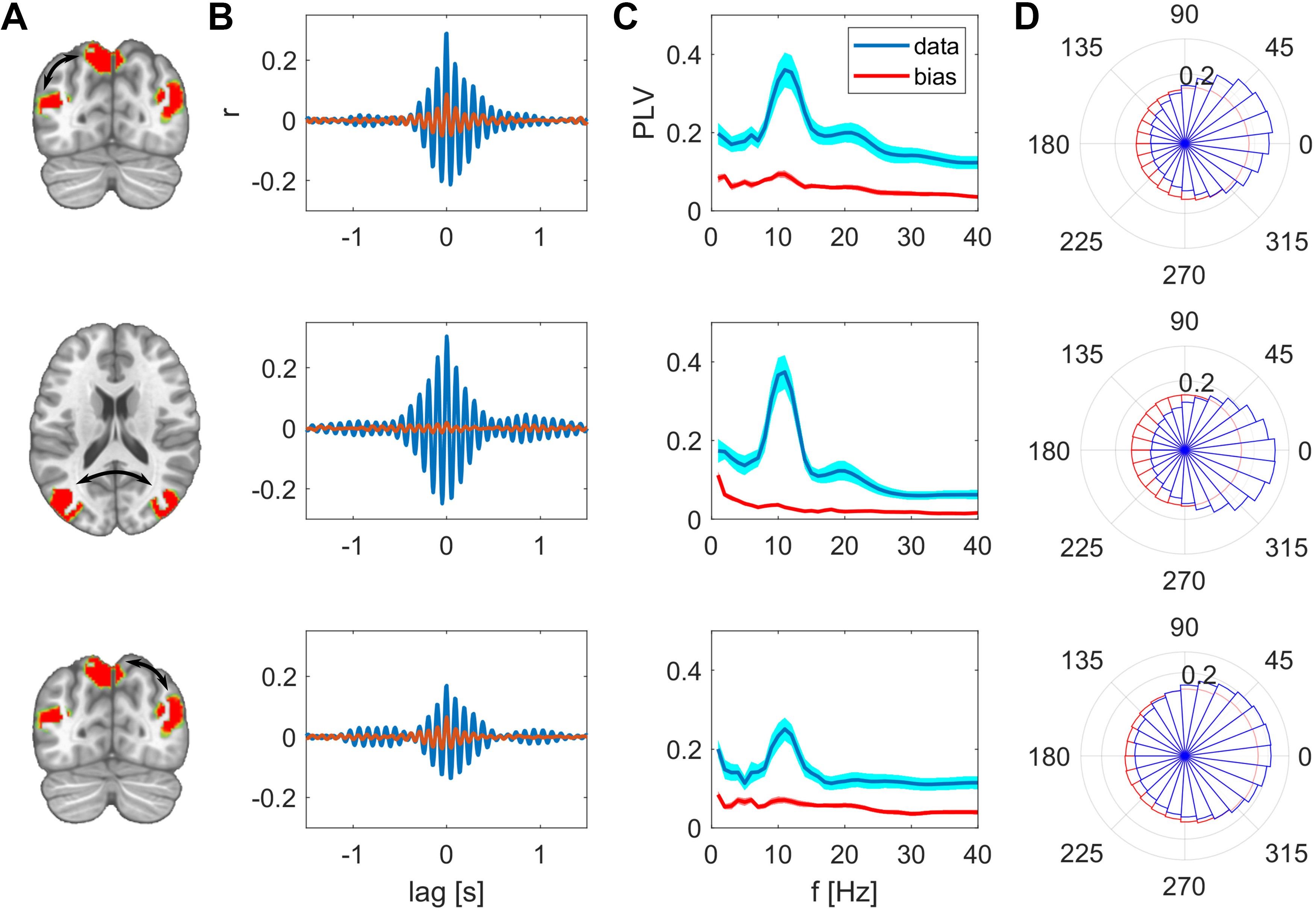
Synchrony between the nodes of the PPC network, uncorrected measures in comparison to spatial leakage bias. ***A***, Nodes of the network, edges are indicated as arrows. ***B***, Cross-correlation between these two nodes, actual (uncorrected) data are shown in magenta, bias in surrogate data in red. ***C***, PLV as function of frequency, group mean ± SEM. ***D***, Polar histograms showing the distribution of phase differences for actual data in blue and surrogate data in red. The radius for each phase bin displays the probability density function estimate of the respective phase differences.

**Figure 5.**
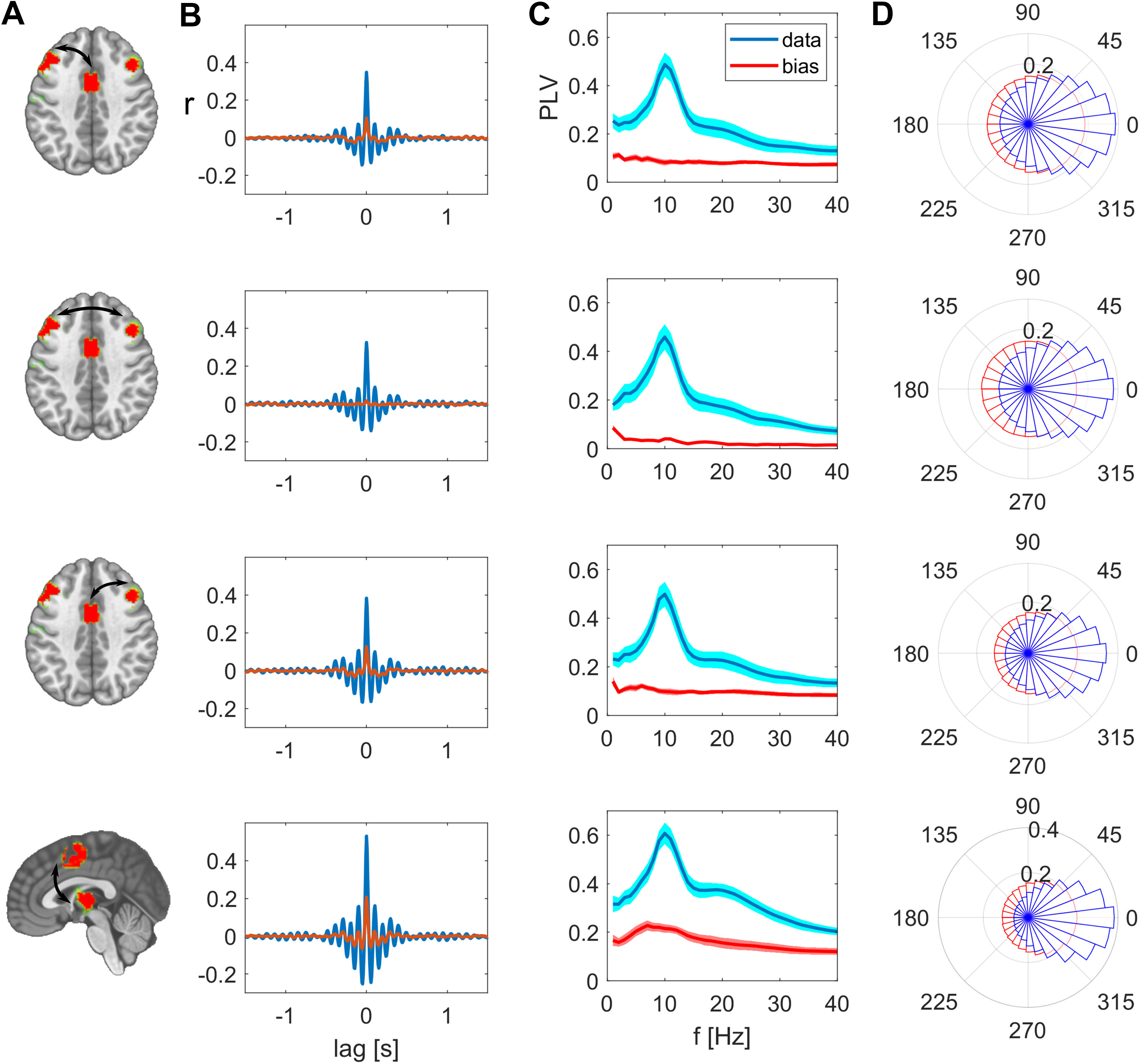
Synchrony between the nodes of the SMA network, uncorrected measures in comparison to spatial leakage bias. ***A***, Nodes of the network, edges are indicated as arrows. ***B***, Cross-correlation between these two nodes, actual (uncorrected) data are shown in magenta, bias in surrogate data in red. ***C***, PLV as function of frequency, group mean ± SEM. ***D***, Polar histograms showing the distribution of phase differences for actual data in blue and surrogate data in red. For every constellation in this network, the most frequent phase difference is zero. The radius for each phase bin displays the probability density function estimate of the respective phase differences.

**Figure 6.**
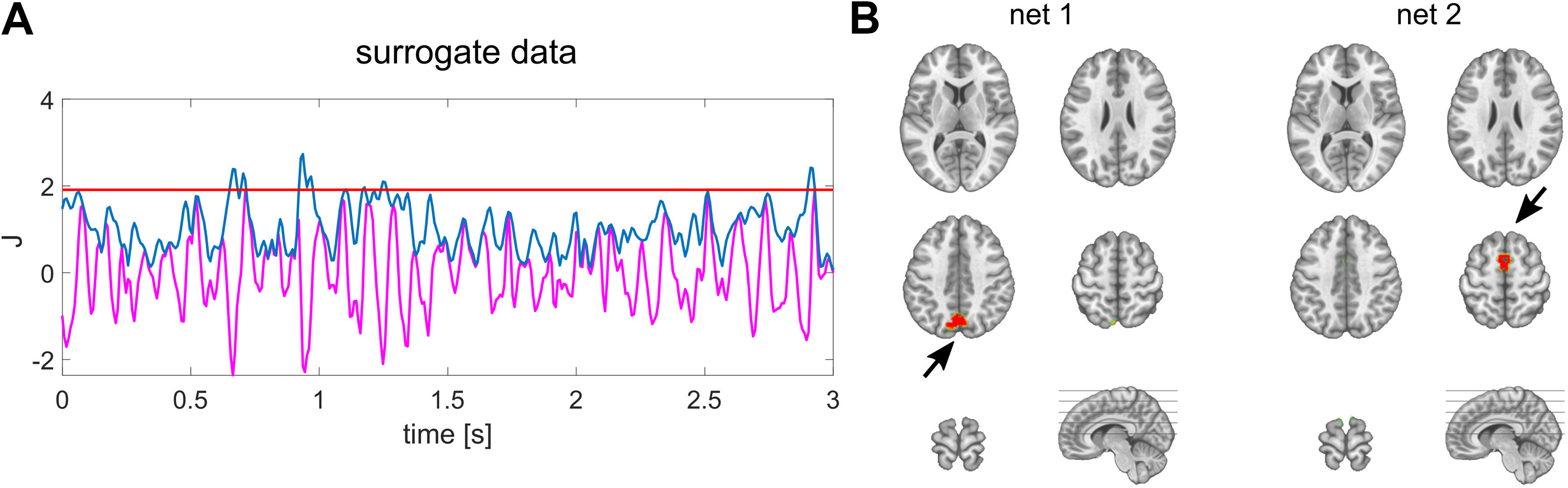
Absence of distant co-varying sites in surrogate data. ***A***, The envelope (blue) of source estimated surrogate data (magenta) is thresholded to define periods of well-pronounced activity within a specific ROI (here PCC). ***B***, No distant local maxima were identified co-varying with the PCC (net 1), or with the SMA (net 2) marked with black arrows.

For direct visual comparison of actual with surrogate data we also show the uncorrected metrics overlaid with the bias estimates in Figures 4, 5. These bias estimates are the higher, the closer a node pair is, but also the lower the spatial resolution between these areas is. For example, the zero-lag correlation peak of the surrogate data are higher for the intrahemispheric pairs (Fig. 4*B*, top and bottom rows), than the bias of the more distant interhemispheric pairs (Fig. 4*B*, middle row). This is analogously the case for the PLV relations in Figure 4*C*. The same applies for comparing the top three rows in Figure 5*C*,*D* for the SMA-based network. The bias resulting from spatial leakage is maximal between SMA and the thalamus, which is plausible given the low spatial resolution in subcortical areas (Fig. 5, bottom row). In addition, spatial leakage is biasing the phase distribution of the surrogate data toward zero, i.e., right in the plots of Figures 4*D*, 5*D*. In other terms, the phase distribution is not circular any more, but biased because of spatial leakage, which is best visible in Figure 5*D*, bottom row (displayed in red). However, for the actual recordings (displayed in blue), the phase bin centered around zero exceeds this bias significantly (*p* values listed in Table 1). Moreover, we did not find any co-varying distant regions in the surrogate data. These results are relevant for ruling a potential source imaging bias out (Fig. 6).

## Discussion

In this work, we investigate synchronous EEG source dynamics between distant brain regions. The functional network patterns we reconstruct revealed spatially well-separated remote brain regions. Investigating the temporally highly resolved phase relations indicating long-range synchronization, we actually observe quasi zero-lag related fluctuations between these distant regions. By comparing these results systematically to surrogate data with identical spatial properties in their source reconstruction, we demonstrate that the observed effects cannot be explained by spatial leakage phenomena.

### Large-scale brain dynamics form briefly stable functional networks

In the reconstruction of functional network patterns, we focused on two key brain regions, i.e., the PCC and SMA. The PCC-based network is composed of bilateral posterior areas of the extrastriate cortex and IPLs. This network resembles the posterior subdivision of the default mode network that was previously reported using MEG recordings (Hipp et al., 2012; Vidaurre et al., 2018). The SMA-based network is composed of the bilateral prefrontal cortex and the thalamus, which are regions associated with the anterior part of the control network (Seeley et al., 2007; Raichle, 2010). We included analyses of thalamic signals because recent work (Krishnaswamy et al., 2017; Seeber et al., 2019) demonstrated the detectability of subcortical activities using EEG source imaging.

However, we did not find a one to one correspondence between the network patterns we observed herein and the M/EEG amplitude correlation-based networks (Brookes et al., 2011; Samogin et al., 2019) that were related to the well-known fMRI resting-state networks (Smith et al., 2009; Raichle, 2010). This discrepancy might stem from the different time-scale of co-variation, i.e., the temporal precision, and coupling measure, which define these functional networks. In this work, phase relations are relevant, since we were aiming for high temporal precision reflecting long-range synchrony. In contrast, in fMRI and MEG/EEG amplitude envelope-based analyses, the temporal alignment on a second scale is sufficient for capturing correlated activities. Phase coherence and amplitude envelope correlation are two types of coupling measures suggested to reflect distinct mechanisms related to different functions (Engel et al., 2013).

We report the nodes of these network patterns synchronizing in brief time intervals, typically in the range of 54–80 ms. These briefly stable epochs and their duration are in good agreement with previously reported time epochs for the EEG microstates (Michel and Koenig, 2018) and transient states derived from MEG recordings using hidden Markov models (HMMs; Vidaurre et al., 2018). However, the HMM states are derived from orthogonalized signals (Colclough et al., 2015) that discard zero-phase relations. EEG microstates are defined as stable topographies. If a particular source network configuration maintains quasi-zero phase relations for a certain period, that necessarily leads to a stable topography of the scalp potential field. Therefore, the brief manifestation of specific quasi zero-lag-related network patterns we describe in this work can be seen as the underlying source dynamics of the microstates.

The temporal dynamics of these briefly stable epochs are characteristic for metastability, i.e., signified by a counterbalance between integrated, i.e., synchronous, and segregated epochs (Tognoli and Kelso, 2014; Deco et al., 2015). In terms of large-scale brain dynamics that means specific nodes of a network pattern are converging into synchrony, i.e., quasi zero-lag relationships, for brief epochs. These integrated, highly synchronous states fall abruptly apart, i.e., segregate, before the next integrated state is established. In that way, it is possible to develop dynamic representations flexibly since distinct states can be installed in different spatial configurations (Tononi and Edelman, 1998; Deco and Kringelbach, 2016; Ju and Bassett, 2020).

### Simultaneous synchronization is present between distant neuronal populations

The fact that we observe spatially well-separated, co-varying sites as network patterns is the first indicator that these distant regions are functionally related at a millisecond time scale. These distant sites are absent when repeating these analyses with surrogate data (Fig. 6). In addition to this spatial assessment, the functional results, e.g., PLVs, we describe herein significantly exceed the bias resulting from spatial leakage, which we derive from surrogate data. As expected, these bias estimates are the higher, the closer two areas are and the lower the spatial resolution at these sites is. Surprisingly, we found the interhemispheric interactions to be higher than the intrahemispheric interactions. Because the distance between respective regions is larger for the interhemispheric than the intrahemispheric pairs, this result cannot be an effect of spatial leakage. These findings together with the cross-correlation peak at zero lag signify genuine simultaneous synchronization between these distant regions.

The finding of long-range, simultaneous synchronization is in line with previous literature showing physiologically relevant, zero-lag relations (Engel et al., 1991; Contreras and Steriade, 1996; Roelfsema et al., 1997) in animals. Recently, a study using intracranial recordings showed interhemispheric zero-lag synchronization in the human brain (O’Reilly and Elsabbagh, 2021). Most of the previous studies investigating synchrony between distant areas were focusing on γ oscillations (>30 Hz) induced by specific tasks (Engel et al., 1991; Roelfsema et al., 1997; Womelsdorf et al., 2007; Siegel et al., 2008; Van Kerkoerle et al., 2014). These γ oscillations were found to facilitate feedforward processing, while mid-frequencies were related to feedback effects from higher areas (Von Stein et al., 2000; Bosman et al., 2012; Van Kerkoerle et al., 2014). Given these differences in task-induced and resting-state signals, it is plausible that the simultaneous fluctuations we describe here represent intrinsic synchrony during minimal sensory input. The finding of quasi zero-phase relations between distant areas might signify functional convergence in these regions during rest, in contrast to sensory-driven time-lagged oscillations induced by a specific task. In that sense, quasi zero-phase relations in distributed areas might relate to the representation and maintenance, rather than the processing of information. This long-range synchronization is briefly stable, not persistently, indicating flexible spatial reconfiguration pertaining to the establishment of particular, re-occurring states. Taken together, we suggest that the balance between temporal stability and spatial flexibility of long-range, simultaneous synchronization patterns is characteristic of the dynamic coordination of large-scale functional brain networks. As such, quasi zero-lag related EEG source fluctuations are physiologically meaningful if spatial leakage is considered appropriately, and should not be excluded in the analysis of functional connectivity using EEG/MEG source imaging.

## Synthesis

Reviewing Editor: Satu Palva, University of Helsinki Decisions are customarily a result of the Reviewing Editor and the peer reviewers coming together and discussing their recommendations until a consensus is reached. When revisions are invited, a fact-based synthesis statement explaining their decision and outlining what is needed to prepare a revision will be listed below. The following reviewer(s) agreed to reveal their identity: Seppo Ahlfors.

This manuscript describes zero-lag synchronous activity between cortical regions, as derived from source estimates of resting state EEG data. By using time-shifted surrogate data, the artefactual contributions from spatial leakage to the zero-lag measures could be subtracted out. The method and observe results provide a means to extend EEG and MEG connectivity analyses to include also zero-lag activity, which is commonly excluded due to methodological concerns. The paper addresses an issue that has important implications to the interpretation of brain-wide activation patterns.

Minor comments:

Schaefer atlas parcellation was used for computing functional connectivity. It should be specified how many parcels did this parcellation have.

The seed regions, the posterior cingulate cortex (PCC) and the supplementary motor area (SMA, could be more clearly indicated in Fig. 1

Line 122: How was the temporal shift in the surrogate data done: how large shifts were used? (Were the shifts substantially longer than the observed duration of stable periods?)

Line 208: “exceeds this bias significantly” - is there a p-value for this?

What was the m-values for Morlet wavelet?

## Author Response

We thank the reviewers for their constructive comments that were tremendously helpful for improving the

manuscript. In the following, we indicate our response in bold font as we did for the changes in the revised

manuscript.

Synthesis Statement:

This manuscript describes zero-lag synchronous activity between cortical regions, as derived from source

estimates of resting state EEG data. By using time-shifted surrogate data, the artefactual contributions from

spatial leakage to the zero-lag measures could be subtracted out. The method and observe results provide a

means to extend EEG and MEG connectivity analyses to include also zero-lag activity, which is commonly

excluded due to methodological concerns. The paper addresses an issue that has important implications to the

interpretation of brain-wide activation patterns.

Minor comments:

Schaefer atlas parcellation was used for computing functional connectivity. It should be specified how many

parcels did this parcellation have.

We used 100 parcels and included this information in the revised manuscript in line 109.

The seed regions, the posterior cingulate cortex (PCC) and the supplementary motor area (SMA), could be

more clearly indicated in Fig. 1

Thank you for pointing that out! We adapted Fig. 1 accordingly, while showing the seed regions in magenta.

Line 122: How was the temporal shift in the surrogate data done: how large shifts were used? (Were the shifts

substantially longer than the observed duration of stable periods?)

This information is indeed relevant; we added following description in line 123f:

"These time shifts were randomly drawn without repeating numbers in the range of 1 and N samples, while

N being the total number of samples.”

Line 208: “exceeds this bias significantly” - is there a p-value for this?

The significance is dependent on the pair ROI. We added two columns in Table 1, which contains the

respective p-values and refer to it in the text, line 210.

What was the m-values for Morlet wavelet?

We added following sentence in line 143 to provide this information:

"At all frequencies, the number of cycles were kept constant meaning the Gaussian kernel of the wavelets

was scaled accordingly, i.e. scale expansion factor of 1.”
